# Parentage assignment in black soldier fly (*Hermetia illucens*) using genotyping-by-sequencing

**DOI:** 10.3389/fgene.2025.1541812

**Published:** 2025-06-12

**Authors:** Guyllaume Dufresne, Catherine Bolduc, Christopher Warburton, Grant Vandenberg, Marie-Hélène Deschamps, Nabeel Alnahhas

**Affiliations:** ^1^ Department of Animal Science, Faculty of Agricultural and Food Sciences, Université Laval, Quebec, QC, Canada; ^2^ Entosystem, Drummondville, QC, Canada

**Keywords:** black soldier fly, parentage assignment, genotyping-by-sequencing, SNP, insects breeding

## Abstract

Genetic selection to optimize economically important traits in black soldier flies (BSF), a major species in the insects as food and feed industry, continues to gain interest. Tracking pedigrees is a prerequisite for generating genetic progress while conserving the genetic variability of traits under selection. However, this is not currently feasible in mass reared insects like BSF. As an alternative, this study identified SNPs informative for parentage assignment (PA) in a commercial and laboratory colony of BSF using genotyping-by-sequencing (GBS). We first established an experimental population of 12 BSF families per colony by randomly mating flies within each family over three generations. DNA was then sequenced from mated pairs and two larvae per pair per generation (n = 288 samples). After SNP calling and filtering, we generated four high-quality SNP subsets containing 192, 118, 72, and 51 SNPs, respectively. PA was conducted using a likelihood-based method across simulated inbreeding rates from 0% to 100%. Compared to known parents, PA accuracy reached 100% across all SNP subsets and inbreeding rates. However, assignment confidence as measured by the log-likelihood (LOD) score decreased significantly as the number of SNPs decreased, though inbreeding had no significant effect on LOD scores. High-confidence assignments to either male or female parents required all 192 SNPs, whereas high-confidence assignments to parent pairs were possible with 118 or 192 SNPs. The identified SNPs provide a valuable resource for developing low-density panels to implement pedigree-based selection and to manage genetic diversity, thereby supporting the development of breeding programs in BSF.

## 1 Introduction

The global population is estimated to exceed nine billion by 2050 (Smith et al., 2024). This growing population will increase the global demand on protein sources (Smith et al., 2024). Over the next decade, global agricultural production is expected to increase by 1.1% annually with livestock and crop production being projected to grow at an annual rate of 1.3% and 1.0%, respectively (OECD, 2024). However, increasing agricultural production is associated with an increase in food loss and food waste which are projected to reach 700 Mt and 1,140 Mt by 2033, respectively (OECD, 2024). Without intervention, food loss and waste could considerably impact food security (Santeramo, 2021) and the environment (Amicarelli et al., 2021).

*Hermetia illucens*, also known as the black soldier fly (BSF), is a dipteran of the Stratiomyidae family originating from subtropical regions of America and can now be found across the globe (Kaya et al., 2021). Due to its short life cycle, high protein and fat content (Barragan-Fonseca et al., 2017), and a capacity to process a variety of organic substrates into protein and biofertilizers (Spranghers et al., 2017), BSF larvae (BSFL) are well-suited for industrial-scale production and upcycling organic waste, lowering the environmental footprint of the agri-food sector, improving its sustainability and providing sustainable entomological protein that can be used to feed livestock, poultry and aquacultural species.

Despite the inherent efficiency of BSFL to convert organic substrates to protein, the improvement of certain traits such as larval weight, survivability and bioconversion is desired. In farm animals, genetic selection is widely used to improve performance traits, reproductive efficiency, and disease resistance, with poultry being a prime example of its effectiveness (Zuidhof et al., 2014). Given its efficiency, genetic selection also holds promise for improving these traits and others in BSF production. For instance, mass selection studies have shown that phenotype-based selection can effectively enhance traits such as larval weight (Facchini et al., 2022) and improve cold resistance (Ma et al., 2024). Mass selection is a breeding method where individuals are selected based on their own phenotype each generation to create the next. This approach is relatively easy to implement, as it relies on phenotypes rather than pedigree tracking, allowing for high selection intensity due to the large number of individuals that can be phenotyped. However, mass selection has important limitations: it can increase inbreeding (Hull et al., 2024) when similar phenotypes are mated, and it is challenging to select multiple traits simultaneously (Slagboom et al., 2024).

In contrast, pedigree-based selection enables breeders to improve multiple traits simultaneously by using a selection index, which aggregates weighted estimated breeding values of traits measured on the same individual. These aggregated values can then be used to rank selection candidates. Pedigree-based selection also allows for more control over inbreeding by designing mating plans in which the mating of related individuals is minimized or avoided. However, this method requires individual identification and pedigree tracking, which are challenging and labor-intensive to implement in insects that are typically raised in large populations (Rikkers et al., 2022). As a result, traditional pedigree-based selection (i.e., pedigree established before birth or hatch), though well-suited for complex trait selection, is cost-prohibitive and logistically challenging to apply in BSF production. As a solution, parentage assignment (PA) using genetic markers (e.g., SNPs or microsatellites) offers an approach to accurately establish pedigrees after birth or hatch without prior knowledge of the exact family structure within a population (Hayes, 2011; Vandeputte and Haffray, 2014). Parentage assignment thus offers a promising alternative to traditional pedigree tracking in insect production systems, retaining the benefits of pedigree-based selection while reducing the labor-intensive management typically required to maintain individual or family identity. Integrating PA into BSF breeding programs could thus help the insect industry address challenges associated with developing sustainable breeding programs for BSF production.

In this work, we aimed to identify SNP markers informative for PA in a laboratory and a commercial population of BSF using Genotyping-by-Sequencing (GBS) to constitute the necessary genomic resources. Several methods have been developed to infer parentage or relatedness from GBS data, each with different strengths depending on the data quality, coverage, and the objective of the analysis. Matrix-based approaches such as kinship estimation using GBS with depth adjustment or KGD (Dodds et al., 2015) estimate pairwise genomic relatedness and are particularly useful for genomic selection and for inferring parentage. Probabilistic frameworks like the one implemented in *AlphaAssign* (Whalen et al., 2019) infer relationships using genotype likelihoods and are robust to ultra-low sequencing coverage (1x – 2x), though they require more complex parameterization and computational steps. Tools like *Sequoia* (Huisman, 2017) can reconstruct multigenerational pedigrees and assign a range of relationship types, including grandparents and half-sibs, making them ideal for complex or natural populations. In contrast, *Cervus* (Kalinowski et al., 2007; Marshall et al., 1998) offers a user-friendly and intuitive solution for assigning parentage in systems where candidate parents are sampled, and genotypes are of sufficient quality. It uses a likelihood-based approach with simulation-derived confidence thresholds to provide straightforward, interpretable assignments, making it particularly well-suited to more structured breeding programs seeking to implement parentage inference without extensive computational efforts.

In this study, we used *Cervus* to conduct parentage assignment analyses because it is widely used and validated tool. Our results show that high PA rates can be achieved in both our subpopulations using fewer than 200 SNPs. To the best of our knowledge, this is the first study to demonstrate the feasibility of PA in BSF using GBS data.

## 2 Materials and methods

### 2.1 Experimental population and sampling

**FIGURE 1:**
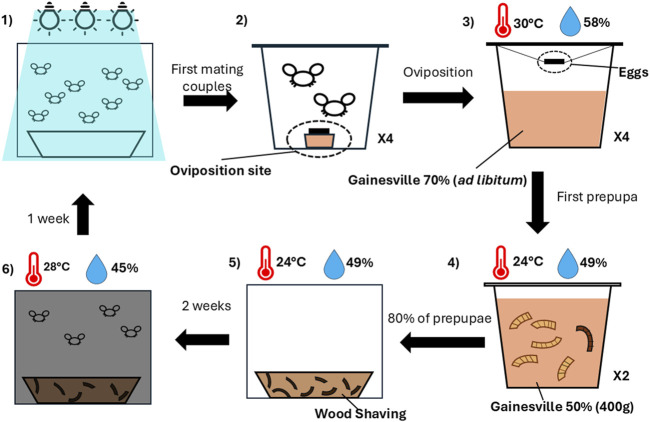
Overview of the rearing phase. (1) Adult flies were exposed to light, and the first four mating pairs were isolated in individual cups containing an oviposition site (2). (3) After oviposition, the eggs were suspended above Gainesville diet (RH 70%) at 30°C and 58% RH. (4) Upon the appearance of the first prepupae, larvae were transferred into 400 g of Gainesville diet (RH 50%) at 24°C and 49% RH. When 80% of the larvae reached the prepupal stage, they were moved to wood shavings and maintained at 24°C and 49% RH for two weeks (5). (6) Pupae were then incubated in a dark room at 28°C and 45% RH for one week, where they emerged as adults ready for mating.

In this study, we sampled two *Hermetia illucens* colonies: a commercial colony (colony ES) maintained at an industrial-scale facility (Entosystem, Drummondville, Québec, Canada), and a laboratory colony (colony UL) maintained at the Laboratoire Aquatique de Recherche en Sciences Environnementales et Médicales (LARSEM, Université Laval, Québec, Canada). The laboratory colony (UL) was established from the commercial colony (ES) in 2020. Since its establishment, the laboratory colony has undergone approximately 52 generations of uninterrupted breeding under controlled conditions. The creation of the experimental population began with 12 families of BSF obtained from and maintained at each source colony (Figure 1). For each family, 200 prepupae were randomly collected from each main colony and placed in aluminum containers (12 × 9 × 5 cm) with orchid potting mix, housed within 900 cm^3^ rearing cages (model 4M1515, BugDorm) for 14 days (colony ES: 24.1°C ± 1.3°C, 48.2% ± 9.7% RH; colony UL: 23.0°C ± 0.8°C, 49.6% ± 3.0% RH).

After 2 weeks or upon the emergence of adults, cages were transferred to a dark room (colony ES: 27.5°C ± 0.4°C, 41.9% ± 7.7% RH; colony UL: 28.1°C ± 0.8°C, 49.6% ± 3.0% RH) for 7 days, where distilled water was sprayed daily. Imagoes were monitored for emergence. Upon sighting adults, rearing cages were transferred under artificial lighting (BSF light; ADSOL, Quebec, Canada), with mating pair formation observed every 10 minutes. For each family, four mating pairs were hand-picked and transferred to individual 960 mL insect pots with mesh covers (model BDPN32–6P, BugDorm). Each pot contained an oviposition medium (colony ES: wood planks; colony UL: corrugated plastic) placed above a cup with an attractant of chicken feed and sugar water (12.5%). Mating pairs were kept under a 12L:12D light cycle, with daily distilled water sprays (colony ES: 26.7°C ± 1.3°C, 42.3% ± 10.2% RH; colony UL: 28.4°C ± 0.9°C, 57.6% ± 5.4% RH). The presence of eggs was checked daily by examining the oviposition medium under a light source. When eggs were observed, the mating pairs were collected, stored in 2 mL tubes, and kept at −20°C until DNA extraction was performed.

The oviposition medium was then suspended inside the insect pot over 200 g of fresh (RH: 70%) Gainesville diet (Hogsette, 1992). Throughout the growth phase, fresh Gainesville diet was given when necessary. Each pot was maintained in a growth chamber (colony ES: 29.6°C ± 0.6°C, 58.4% ± 6.4% RH; colony UL: 29.2°C ± 0.9°C, 57.6% ± 2.8% RH) and daily observation of hatched eggs was performed using a stereomicroscope. Five days-old neonates were separated from the oviposition medium using a fine mesh strainer, rinsed using lukewarm tap water, dried on an absorbent paper and total biomass was measured. For each mating pair, 25 randomly selected larvae were weighed to estimate individual mass. The larvae from the two pots having the highest biomasses were kept. A total of 250 larvae were transferred into 400 g of homogenized Gainesville diet (70% RH) and returned to the growth chamber for an additional 5 days. Four sibling larvae (minimum of 40 mg of weight) were randomly collected, placed in 2 mL tubes and stored at −20°C until DNA extraction.

At 10 days, total larval biomass was measured again, and larvae were transferred in 400 g of fresh Gainesville diet (70% RH). Upon observing the first prepupa, larvae were counted manually and transferred into 300 g of Gainesville diet (50% RH). When at least 80% reached the prepupal stage within each family, 200 prepupae from the pot with the highest biomass were selected to establish the next-generation. This standardized protocol of mating, rearing, and sampling (Figure 1) was repeated for three generations at each colony to constitute the experimental population.

### 2.2 DNA extraction, library preparation and sequencing

DNA extraction, quantification, normalization, and GBS library preparation were carried out at the Plateforme d'Analyses Génomiques, Institut de Biologie Intégrative et des Systèmes (Université Laval, Quebec City, Quebec, Canada). In brief, DNA was extracted from the thorax of adult male and female flies and from whole larvae stored at −20°C, using the E-Z 96™ Tissue DNA Kit (Omega Bio-Tek) according to the manufacturer’s instructions. DNA concentrations were measured with the Quant-iT dsDNA Assay (broad range; Fisher Scientific) and normalized to 10 ng/μL.

Sequencing libraries were subsequently prepared using the 3D-GBS method (de Ronne et al., 2023). This protocol utilizes three restriction enzymes—PstI, NsiI, and MspI—with 100 ng of genomic DNA per sample, digested by the enzyme mix and ligated with sample-specific barcoded adapters. The 5′ adapter overhangs align with the PstI and NsiI enzyme cuts, while the 3′ adapter overhangs align with those produced by MspI (de Ronne et al., 2023). Pooled individual libraries were size-selected (100–350 bp) using a BluePippin apparatus (Sage Science, MA, United States), followed by a 12-cycle PCR amplification and a cleanup step with Ampure beads (1X ratio; Beckman).

The final libraries were quality-checked on a Bioanalyzer 2,100 using High Sensitivity chips (Agilent) and quantified with a Qubit Fluorometer (Fisher Scientific). Libraries were then shipped for paired-end sequencing (150 bp, 35M reads) on the Illumina NovaSeq X sequencer at Centre d’expertise et de services Génome Québec (Montreal, Quebec, Canada).

### 2.3 Adapter trimming and read preprocessing

We began by removing adapter sequences from the raw FASTQ files and trimming reads using *Cutadapt* (Martin, 2011). Parameters were set to allow a maximum error rate of 0.2 (option -e 0.2) and a minimum read length of 50 bp (option -m 50). Next, we used the process_*radtags* module of *Stacks* (Catchen et al., 2013) to separate samples (demultiplexing), remove reads with uncalled bases (option -c), discard low-quality reads (option -q), rescue barcodes and RAD-tag cut sites (option -r), and limit barcode mismatches to a maximum of two (option--barcode_dist 2). Finally, all reads were trimmed to a consistent length of 100 bp (option -t 100). Following demultiplexing, *fastqc* (Andrews, 2010) was used to assess the quality of the reads for each sample.

### 2.4 Read alignment

The processed reads were then aligned to the black soldier fly genome from Ensembl (*Hermetia illucens*, assembly *iHerIll2.2. curated.20191125*, accession GCA_905115235) using the BWA-MEM2 alignment algorithm (Vasimuddin et al., 2019). The parameters included a minimum seed length of 19 (option -k 19), discarding exact matches that had more than 500 occurrences in the genome (option -c 500), a gap open penalty of 0 (option -O 0,0), and a gap extension penalty of two (option -E 2,2). We only retained alignments with a score greater than 10 (option -T 10). All other parameters of the aligner were at default values. The output alignment files (in SAM format) were then converted to BAM format, and these files were sorted using SAMtools (Li et al., 2009). The total length of this assembly is 1.0 Gb and is organized into seven chromosomes.

### 2.5 SNP calling and genotyping

We used the *Gstacks* module of *Stacks* (Catchen et al., 2013) version 2.68 to identify loci from paired-end reads aligned to the genome, detect single nucleotide polymorphisms (SNPs), and call genotypes for individual samples. Parameters included a maximum soft-clipping level of 10% of the read length (option --max-clipped 0.1), unpaired reads were removed (option --rm-upaired-reads), with all other parameters left at default values.

### 2.6 Population statistics

For exploratory population analysis, we used the *populations* module of *Stacks* (Catchen et al., 2013) version 2.68. This module computed basic population statistics. Loci included in these calculations were required to be present in at least two populations (option -p 2) and observed in at least 60% of samples in each population. We filtered to retain only SNPs with a MAF of at least 5% (option --min-maf 0.05) and a minor allele count of at least four (option --min-mac 4). SNPs with observed heterozygosity above 0.5 (--max-obs-het 0.5) and SNPs that were not in HWE (P < 0.05) were excluded. Only one SNP per locus was exported to avoid strong linkage disequilibrium (--write-single-snp). Inbreeding coefficients (F_IS_) and fixation index (F_ST_) were also calculated using the *populations* module in *Stacks* (Catchen et al., 2013), based on observed and expected heterozygosity and using kernel-smoothing (options --smooth, --smooth-fstats, and --smooth-popstats), which apply a Gaussian-weighted kernel smoothing across a sliding window along the genome without altering raw estimates to facilitate detection of genomic trends.

### 2.7 Principal component analysis and analysis of molecular variance

SNPs exported from *Stacks* in Section 2.6 were further filtered using the *dartR* package of R (Gruber et al., 2018). SNPs were retained if they were genotyped in at least 95% of samples (SNP-wise call rate of 95%) and if each sample was genotyped at a minimum of 90% of loci (sample-wise call rate of 90%). Only SNPs with a MAF ≥ 5% were retained. We then conducted a principal component analysis (PCA) on the remaining SNPs using the same R package to illustrate the genetic similarities and differences between the two subpopulations of the study. Finally, an analysis of molecular variance (AMOVA) was also conducted on the same set of SNPs to statistically test if the differentiation between the two experimental subpopulations was significant. AMOVA was conducted using the *poppr. amova* function from the *poppr* package of R (Kamvar et al., 2014). The analysis was based on a quasi-Euclidean genetic distance matrix, allowing for missing data. The total genetic variance was partitioned into three components: variation between populations, among individuals within populations, and within individuals. Statistical significance of the variance components was assessed using a permutation test with 999 replicates implemented via the *randtest* function from the same package.

### 2.8 SNP filtering

**FIGURE 2:**
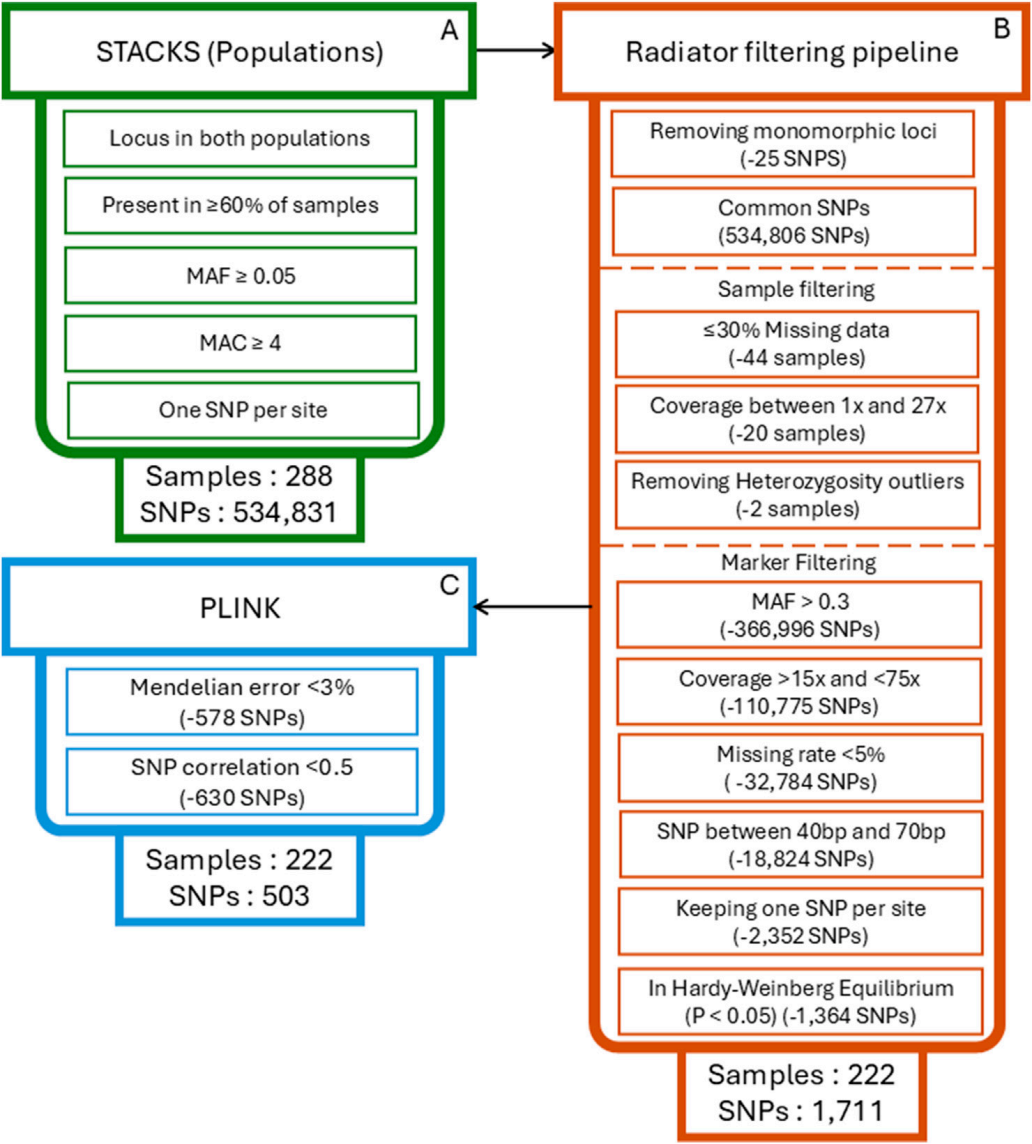
Bioinformatics pipeline used to produce the reduced high-quality SNP sets for parentage assignment. **(A)** SNP calling and initial filtering using the populations module of Stacks. **(B)** SNP filtering pipeline implemented in Radiator, including sample filtering and marker filtering steps. **(C)** Additional filtering in PLINK to remove Mendelian errors and SNPs with high correlations.

The *populations* module of *Stacks* (Catchen et al., 2013) does not offer sufficient flexibility for more specific variant filtering and identifying of SNPs suitable for PA. For this reason, we conducted a new run of *populations* to export the called SNPs into a variant call format (VCF) file with the same parameters indicated in Section 2.6, except that no filtering based on observed heterozygosity or HWE was applied (Figure 2A).

The exported VCF file contained 288 samples and a total of 534,831 SNPs. For PA analyses, a high-quality reduced set of informative SNPs is required. To obtain this set of markers (Figure 2B), the VCF file was filtered using the *filter_rad* function of the R package *Radiator* (Gosselin et al., 2020). First, we removed monomorphic markers (n = 25 SNPs) and kept only markers common in samples from both colonies (n = 534,806 SNPs). We then removed samples with more than 30% of missing markers (n = 44 samples), with median coverage values outside of 1 to 27x (n = 20 samples) or with heterozygosity values outside of 0.14–0.61 (n = 2 samples) because values out of these ranges were identified by *Radiator* as outliers. Then, we removed markers with a MAF lower than 0.3 (n = 366,996 SNPs), an average coverage less than 15 or more than 75 reads (n = 110,775 SNPs), a missingness greater than 5% (n = 32,784 SNPs), and not situated between the 40th and 70th bp on the RAD locus (n = 18,824 SNPs). On each RAD locus, we only kept one SNP to avoid strong LD, which led to the removal of an additional 2,352 SNPs. We also removed SNPs that were in Hardy-Weinberg disequilibrium (P < 0.05, n = 1,364 SNPs).

Following this filtering, 222 samples and 1,711 SNPs remained, which we exported into a new VCF file. We removed SNPs with a Mendelian error rate higher than 3% from this file using Plink V1.9 (Chang et al., 2015) based on known parent–offspring trios (option --me 1 0.03) and a total of 1,133 SNPs remained. Parentage assignment tools assume loci are independent. To meet this requirement, we removed from the remaining SNPs those with an *r* ^2^ greater than 0.5 in a window of 50 bp in length shifting by five bp using Plink (option --indep-pairwise 50 5 0.5) which further removed 630 SNPs. At this stage, the filtered dataset contained 222 samples and 503 SNPs (Figure 2C).

### 2.9 Creating the final SNP subsets for parentage assignment

PA requires SNPs with high informativeness across populations that are also not biased toward rare or population-specific variants. These markers should also be as evenly distributed across all chromosomes as possible to minimize genomic clustering and to enhance the feasibility of PA. Consequently, from the remaining SNPs, four final subsets (Supplementary Tables S1–S4) were created by further filtering based on MAF >0.40 (n = 192 SNPs), >0.45 (n = 118 SNPs), >0.47 (n = 72 SNPs), and >0.48 (n = 51 SNPs). These four datasets were recoded using Plink (option --recode 12) and used for further analyses. In these datasets, a total of 47 couples (n = 19 and 28 couples from the ES and UL colonies, respectively) with 75 larvae (n = 23 and 52 from the ES and UL colonies, respectively) remained and were used for PA analyses (Supplementary Tables S5–S7).

### 2.10 Parentage assignment (PA) analysis

Parentage assignment analyses were conducted using *Cervus*, a software that applies a likelihood-based approach to account for possible genotyping errors in parents and offspring (Kalinowski et al., 2007; Marshall et al., 1998). Using *Cervus* is a three-step process. In step 1, allele frequencies are computed from genotypes. In step 2, a simulation of PA is conducted on a large population of offsprings (n = 10,000) to determine the critical log-likelihood (LOD) scores (C-LOD) that are then used to determine the confidence level of the assignment. This step takes into account the allele frequencies computed in step 1, the number of candidate parents (n = 47), the proportion of sampled parents (set to 1.0 in all analyses), the proportion of typed loci (set to 0.95 in all analyses), the proportion of mistyped loci (set to 0.05 in all analyses) and the minimum number of typed loci (set to 50% of the number of SNPs used in the analyses). These parameters are consistent with the filtering thresholds applied to the dataset in Section 2.8. The 95% proportion of typed loci reflects the ≤5% missing data allowed per SNP in our filtering, while the 5% mistyping rate accounts for the potential genotyping errors inherent to GBS-based SNP datasets, such as allele dropout or base-calling inaccuracies. The C-LOD scores, which range from −999.0 (indicating a very high-power marker set) to +999.0 (indicating a very low power marker set), are used to determine the most likely parent or parent pair of each offspring at both relaxed (80%) and strict (95%) confidence levels. In the third and final step, the PA is conducted incorporating results from the first two steps with an offsprings file indicating the list of potential or candidate parents for each offspring to be tested. *Cervus* computes the probability of a candidate parent or a parent pair being the true parent or parent pair of the tested individual (P1) and the probability of the candidate parent or parent pair not being the true parent or parent pair of the tested individual (P2). Then, it takes the log of the ratio of P1/P2 as the assignment LOD score (A-LOD). This ratio indicates how likely candidate parent is to be the true parent. The A-LOD score is then compared to the C-LOD scores corresponding to each confidence level (80% and 95%). When the A-LOD exceeds the C-LOD determined at the strict (95%) or relaxed (80%) confidence level, the tested individual is assigned to the candidate parent or parent pair at that confidence level.

In this study, we conducted PA analyses using the four above-mentioned final SNP subsets. Given the method that we used to constitute the experimental population, parents within a given family were related. To account for this relationship and to understand its impact on the assignment analysis, we performed parentage analysis simulations with the following parameters: parents from each sex were set as relatives to parents from the opposite sex with a relatedness coefficient of 0.5 (i.e., full sibs) in *Cervus* (the “Relatives” tab). As mating related individuals leads to inbred offsprings, we repeated each simulation with the following rate of inbreeding: 0.0, 5.0, 10.0, 25.0, 50.0, and 100.0% (the “Inbreeding” tab in *Cervus*) to evaluate the effect of increased inbreeding on the capacity of the four SNP subsets to correctly and confidently assign tested larvae to their parents.

Each offspring was only compared to parents from the same colony (commercial or laboratory) and generation, ensuring that offspring were not tested against parents from a different colony or generation as the colony of origin and the generation were known. Assignment rates were calculated by comparing *Cervus*-assigned parents to the true known parents and were reported as a percentage. As for the confidence level of assignment, according to the user guide, *Cervus* determines it through the simulation step described above. In more detail, during the simulation step, parentage analysis is performed exactly as it would be with real genotype data, but with the key difference that the true parent of each simulated offspring is known. *Cervus* uses these simulations to compare two distributions: the LOD scores for cases where the most likely candidate parent is the true parent, and the LOD scores for cases where the most likely candidate is not the true parent. The confidence level for assignment is then based on how well these two distributions can be separated. Specifically, *Cervus* defines confidence as the proportion of candidate parents with an A-LOD score exceeding a given C-LOD threshold who are true parents. For instance, if the simulations determine a C-LOD value such that 95% of A-LOD scores of candidate parents exceeding this value are true parents and only 5% are false positives, then this C-LOD value corresponds to a 95% confidence threshold. Using this same method, *Cervus* determines two confidence thresholds: relaxed (80% confidence) and strict (95% confidence). If a parent or a parent pair does not meet at least the relaxed threshold, they are unassigned. However, *Cervus* output them as the most likely or not the most likely parent or parent pairs. This creates four confidence classes: Strict, relaxed, unassigned most likely, and unassigned not most likely parent or parent pair.

### 2.11 Statistical analysis

We evaluated the effect of the colony of origin (laboratory vs. commercial) on the population genetic parameters including observed and expected heterozygosity, nucleotide diversity (Pi), and on F_IS_ using a Wilcoxon test as implemented in the *compare_means* function of the *ggpubr* package of R.

To assess the impact of the number of SNPs included in the PA analyses (n = 4 SNP subsets) on A-LOD scores, an ANOVA was performed using the *stat_compare_means* function of the *ggpubr* package followed by t-tests to identify significant differences between the levels of this experimental factor. These t-tests were conducted using the *geom_pwc* function of *ggpubr* and Bonferroni adjusted P-values were reported. In this analysis, the 192 SNP set was used as the reference group.

## 3 Results

### 3.1 Population statistics

The effective per-sample coverage computed by the *Gstacks* module ranged from 1.2 to 75.8x with an average (± standard deviation) across all samples of 12.2x ± 9.2x. From an initial ∼1.27 million loci assembled by *Stacks* and after initial filtering as described in Section 2.6, 59,244 loci passed quality control. These loci covered ∼13 million genomic bases, resulting in 32,635 SNPs available for population genetic analyses. The loci had a consistent average size of ∼207 bp. Further filtering as described in Section 2.7 reduced the number of SNPs to 4,727.

**FIGURE 3:**
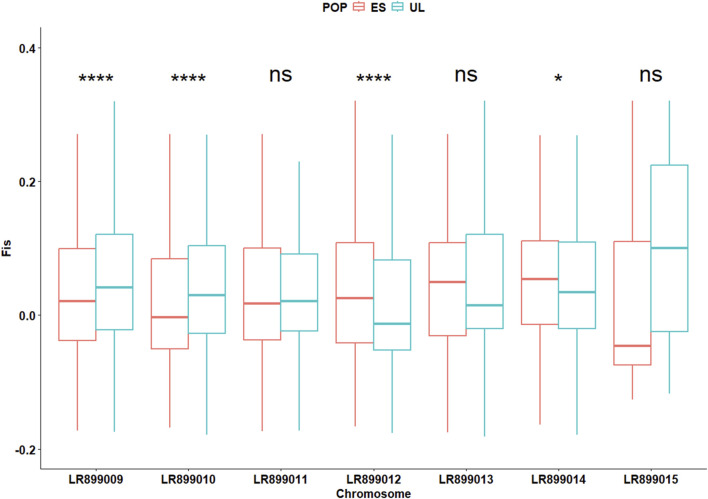
Chromosome-specific inbreeding coefficients (Fis) in commercial (ES) and laboratory (UL) colonies. Significance levels are based on Wilcoxon test (n = 4,727 SNPs): NS (P > 0.05), * (P < 0.05), ** (P < 0.01), *** (P < 0.001).

**FIGURE 4:**
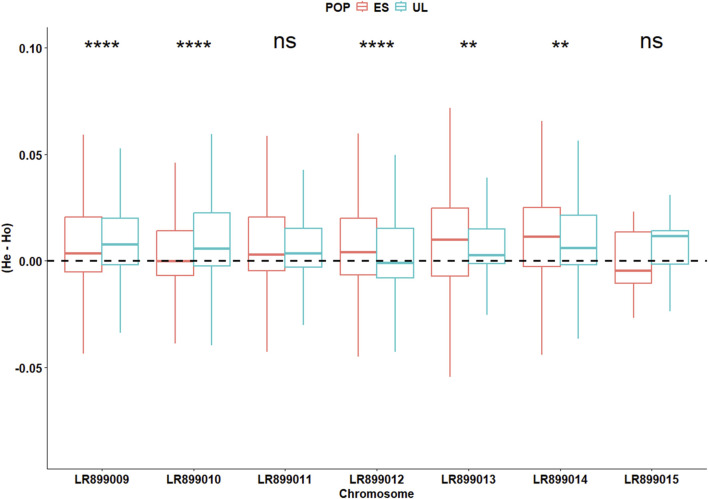
Chromosome-specific deviation between the expected (H_e_) and the observed (H_o_) heterozygosity in commercial (ES) and laboratory (UL) colonies. Significance levels are based on Wilcoxon test (4,727 SNPs): NS (P > 0.05), * (P < 0.05), ** (P < 0.01), *** (P < 0.001).

**FIGURE 5:**
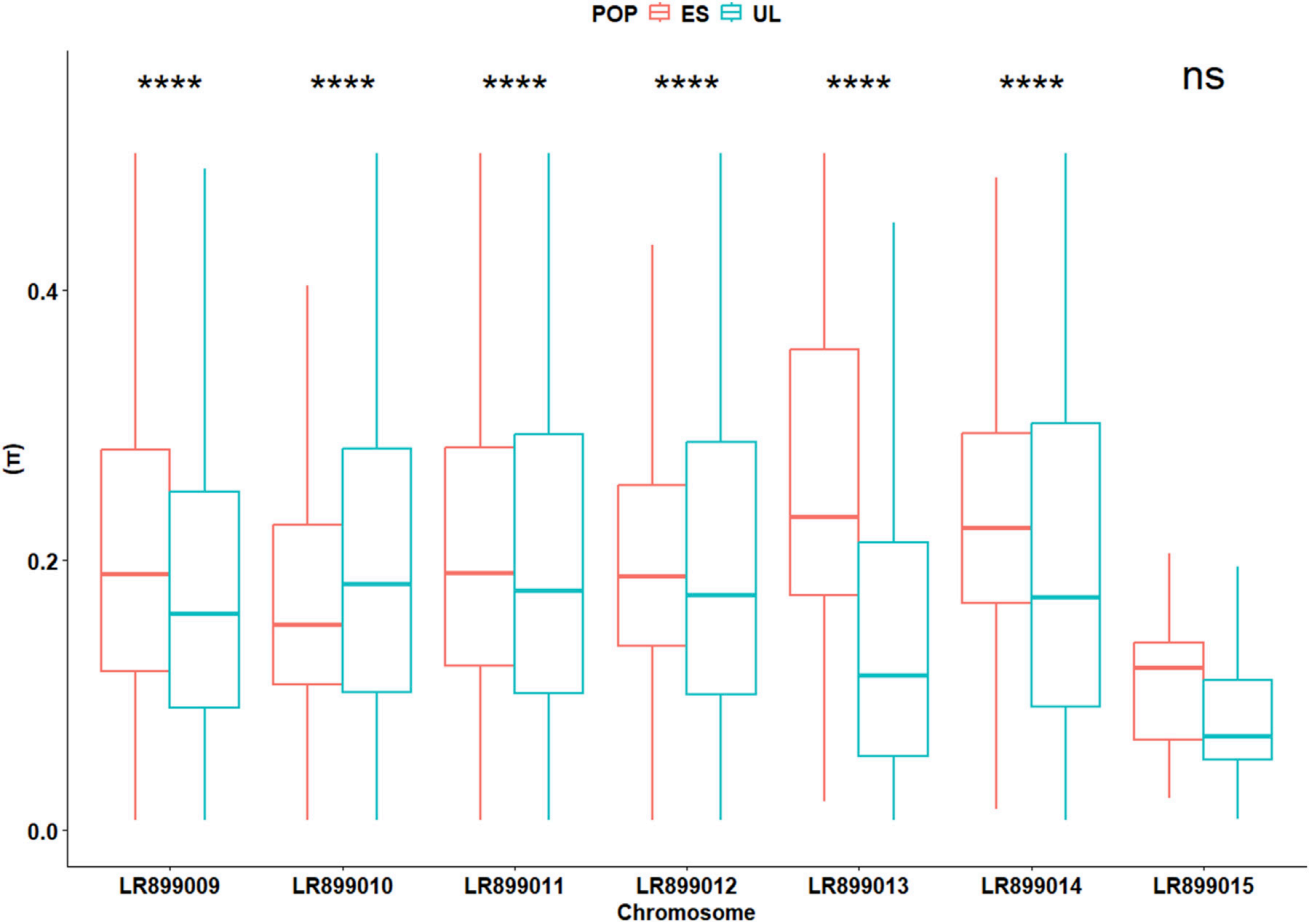
Chromosome-specific nucleotide diversity (Π) in commercial (ES) and laboratory (UL) colonies. Significance levels are based on Wilcoxon test (4,727 SNPs): NS (P > 0.05), * (P < 0.05), ** (P < 0.01), *** (P < 0.001).

In the initially filtered SNP dataset (n = 4,727 SNPs), MAF distributions visualized separately by colony (Supplementary Figure S1), generation (Supplementary Figure S2), and by colony × generation combination (Supplementary Figure S3) did not show marked skewness toward high-frequency alleles. The average locus-wise F_ST_ for the pair of subpopulations was 0.028, indicating low but detectable differentiation. Overall, Wilcoxon test revealed slight but significant differences (P < 0.001) between the two subpopulations in terms of F_IS_ (0.030 vs. 0.035 for ES and UL, respectively), Pi (0.21 vs. 0.19 for ES and UL, respectively), H_o_ (0.20 vs. 0.19 for ES and UL, respectively), and H_e_ (0.21 vs. 0.19 for ES and UL, respectively). However, chromosome-level analyses of these parameters revealed inconsistent differences between the two subpopulations. Across different chromosomes, F_IS_ varied significantly but in different directions between the two subpopulations, indicative of a differing levels of per chromosome heterozygote deficit between them (Figure 3). This inconsistent pattern of per chromosome differences was also observed for the deviation between H_e_ and H_o_ (Δ_Het_, Figure 4), and for Pi (Figure 5).

**FIGURE 6:**
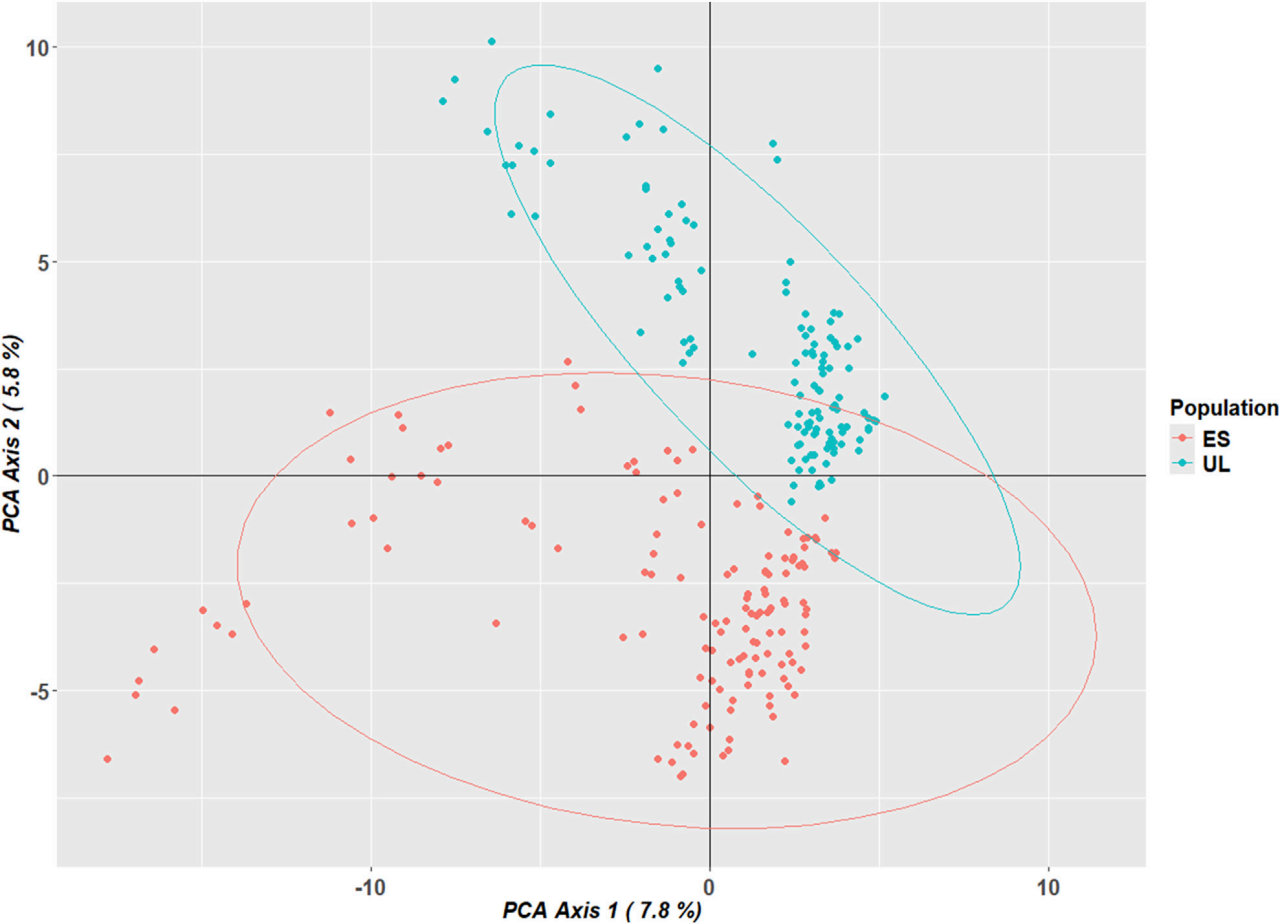
Principal Component Analysis (PCA) of 264 samples based on 4,727 SNPs. Individuals are colored according to their colony of origin. Ellipses represent 95% confidence intervals around each population cluster. The first principal component (PC1) explains 7.8% of the total genetic variation, while the second PC explains 5.8%. Separation between commercial (ES) and laboratory (UL) populations indicates moderate genetic differentiation.

Principal component analysis of the 4,727 filtered SNPs (Figure 6) demonstrated a visible separation between the two subpopulations along the first two principal components, consistent with the genetic differentiation observed above. Despite the relatively low F_ST_ value, the PCA suggests a degree of genetic structure.

To further quantify the genetic differentiation revealed by the PCA, an AMOVA was conducted on the same SNP dataset (Table 1). The results indicated that 5.58% of the total genetic variation was attributable to differences between the laboratory and commercial subpopulations (P = 0.001), 13.67% was explained by variation among individuals within subpopulations (P = 0.001), and the majority of genetic variance (80.74%) was found within individuals (P = 0.001). Although the between-population component was relatively small compared to within-population variation, its statistical significance corroborates the genetic structure observed in the PCA.

**TABLE 1:** Results of AMOVA showing genetic variance partitioned among and within black soldier fly subpopulations^a^.

Source of variation	DF	SS	Component	%	P-value
Between Populations	1	14,440.24	51.05	5.58	0.001
Among Individuals (Within Pop)	262	259,180.64	125.15	13.67	0.001
Within Individuals	264	195,079.31	738.93	80.74	0.001
Total	527	468,700.19	915.144	100	–

^a^ DF: degrees of freedom, SS: sum of squares, Component: variance component, %: percentage of the total variance in the dataset captured by the respective components, P-value: P-value of the permutation test based on 999 permutations.

### 3.2 Characteristics of marker sets used for parentage assignment

**FIGURE 7:**
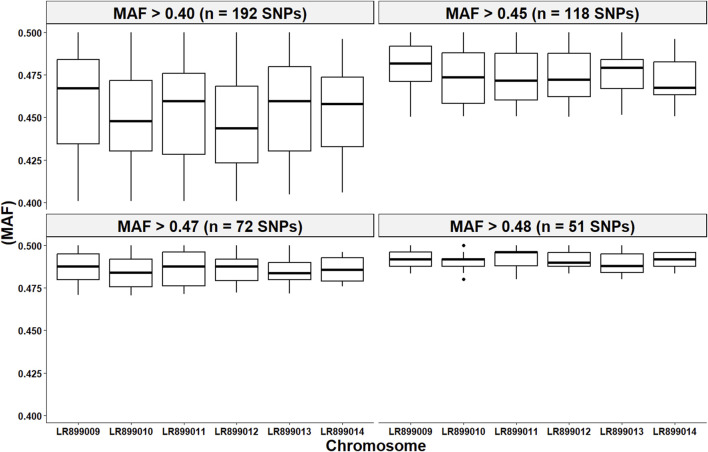
Per chromosome distribution of the minor allele frequency (MAF) from the four high-quality SNP subsets utilized in the parentage assignment analyses. Each panel in this figure represents a subset of SNPs that passed a MAF filtering threshold between >0.40 and >0.48.

Figure 7 illustrates the distribution of retained high-quality SNPs across chromosomes for each of the four marker subsets used in parentage assignment. As expected, increasing the MAF threshold reduced the number of SNPs per dataset (Table 2), resulting in subsets of 192, 118, 72, and 51 SNPs. Despite this reduction in marker number, key genetic parameters remained consistent across SNP subsets. The proportion of typed loci, mean H_e_, mean H_o_, and polymorphism information content (PIC), as calculated by C*ervus*, were stable regardless of SNP density. This indicates that the most informative markers were retained even under stringent MAF filtering. While non-exclusion probabilities (NEP), which estimate the probability that an unrelated individual cannot be excluded as a parent, increased slightly as the number of SNPs decreased, they remained consistently low across all subsets.

**TABLE 2:** Characteristics of the final marker sets used in the parentage assignment analyses.

Parameter	Marker subset			
	MAF >0.40	MAF >0.45	MAF >0.47	MAF >0.48
Individual genotyped	222	222	222	222
Number of loci used	192	118	72	51
Mean proportion of typed loci	0.97	0.97	0.97	0.97
Mean expected heterozygosity	0.49	0.5	0.5	0.5
Mean observed heterozygosity	0.46	0.47	0.47	0.47
Mean polymorphic information content	0.37	0.37	0.37	0.37
Non-exclusion probability 1st parent	1.27E-11	1.60E-07	6.81E-05	1.11E-03
Non-exclusion probability 2nd parent	6.58E-18	2.42E-11	3.20E-07	2.53E-05
Non-exclusion probability parent pair	4.45E-28	1.29E-17	4.79E-11	5.00E-08

### 3.3 Parentage assignment

The effect of the simulated inbreeding rate on C-LOD scores was minimal, whereas the number of SNPs included in the simulations had a considerable effect (Table 3).

**TABLE 3:** The simulation-determined critical log-likelihood (LOD) scores^a^ of parentage assignment according to the number of SNPs included in the analyses and to the simulated inbreeding rate.

Parent	SNPs	Confidence (%)	Inbreeding (%)					
			0	5	10	25	50	100
Male	192	80	−999.0	−999.0	−999.0	−999.0	−999.0	−999.0
	118	80	−999.0	−999.0	−999.0	−999.0	−999.0	−999.0
	72	80	11.5	11.0	10.7	10.1	9.6	7.5
	51	80	12.6	11.4	10.9	10.6	10.4	10.7
	192	95	−999.0	−999.0	−999.0	−999.0	−999.0	−999.0
	118	95	14.6	15.3	14.6	14.2	13.4	8.8
	72	95	15.6	14.2	14.8	14.8	15.0	14.5
	51	95	13.5	14.6	15.7	14.1	14.2	13.5
Female	192	80	−999.0	−999.0	−999.0	−999.0	−999.0	−999.0
	118	80	−999.0	−999.0	−999.0	−999.0	−999.0	−999.0
	72	80	11.2	10.7	10.6	10.1	9.5	7.9
	51	80	10.9	11.0	11.1	10.3	10.9	10.3
	192	95	−999.0	−999.0	−999.0	−999.0	−999.0	−999.0
	118	95	14.5	15.1	14.6	14.1	13.5	−999.0
	72	95	14.1	14.7	15.2	14.6	14.9	14.4
	51	95	12.3	13.5	14.0	14.8	13.6	14.2
Parent pair	192	80	−999.0	−999.0	−999.0	−999.0	−999.0	−999.0
	118	80	−999.0	−999.0	−999.0	−999.0	−999.0	−999.0
	72	80	−999.0	−999.0	−999.0	−999.0	−999.0	−999.0
	51	80	16.9	17.3	17.7	18.1	19.6	19.7
	192	95	−999.0	−999.0	−999.0	−999.0	−999.0	−999.0
	118	95	−999.0	−999.0	−999.0	−999.0	−999.0	−999.0
	72	95	20.0	10.9	20.6	21.1	22.5	23.8
	51	95	22.0	24.9	31.5	26.7	26.0	28.3

^a^ Values of critical LOD scores (C-LOD) vary between −999.0 (very high-power set of markers) to 999.0 (very low power set of markers).

At the strict (95%) confidence level, *Cervus* reported a C-LOD score of −999.0 when 192 SNPs were used, indicating a marker set with such high discriminatory power that no finite threshold could be estimated in the simulation. As the number of SNPs decreased, the C-LOD values increased and became positive, reflecting a loss of statistical power and making high-confidence assignments more difficult to achieve.

At the relaxed (80%) confidence level, C-LOD scores remained negative for larger SNP sets but became positive in the smallest sets (72 and 51 SNPs), consistent with the reduced informativeness of these marker sets. A similar trend was observed in parent pair assignments, where C-LOD values were negative or near zero for the larger panels and became positive only when using 51 SNPs.

**FIGURE 8:**
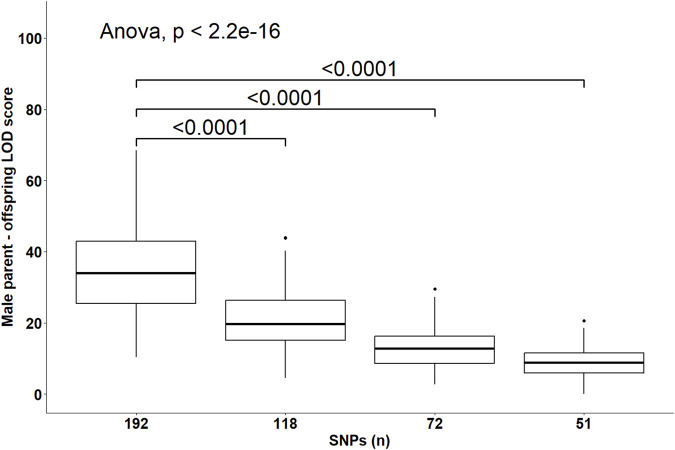
Distribution of the log-likelihood (LOD) scores of assignments to the male parent according to the number of SNPs included in the parentage assignment analysis. Brackets and their Bonferroni-corrected P-values indicate a significant difference in LOD scores in the 118, 72 and 51 SNPs datasets compared to the 192 SNPs dataset.

**FIGURE 9:**
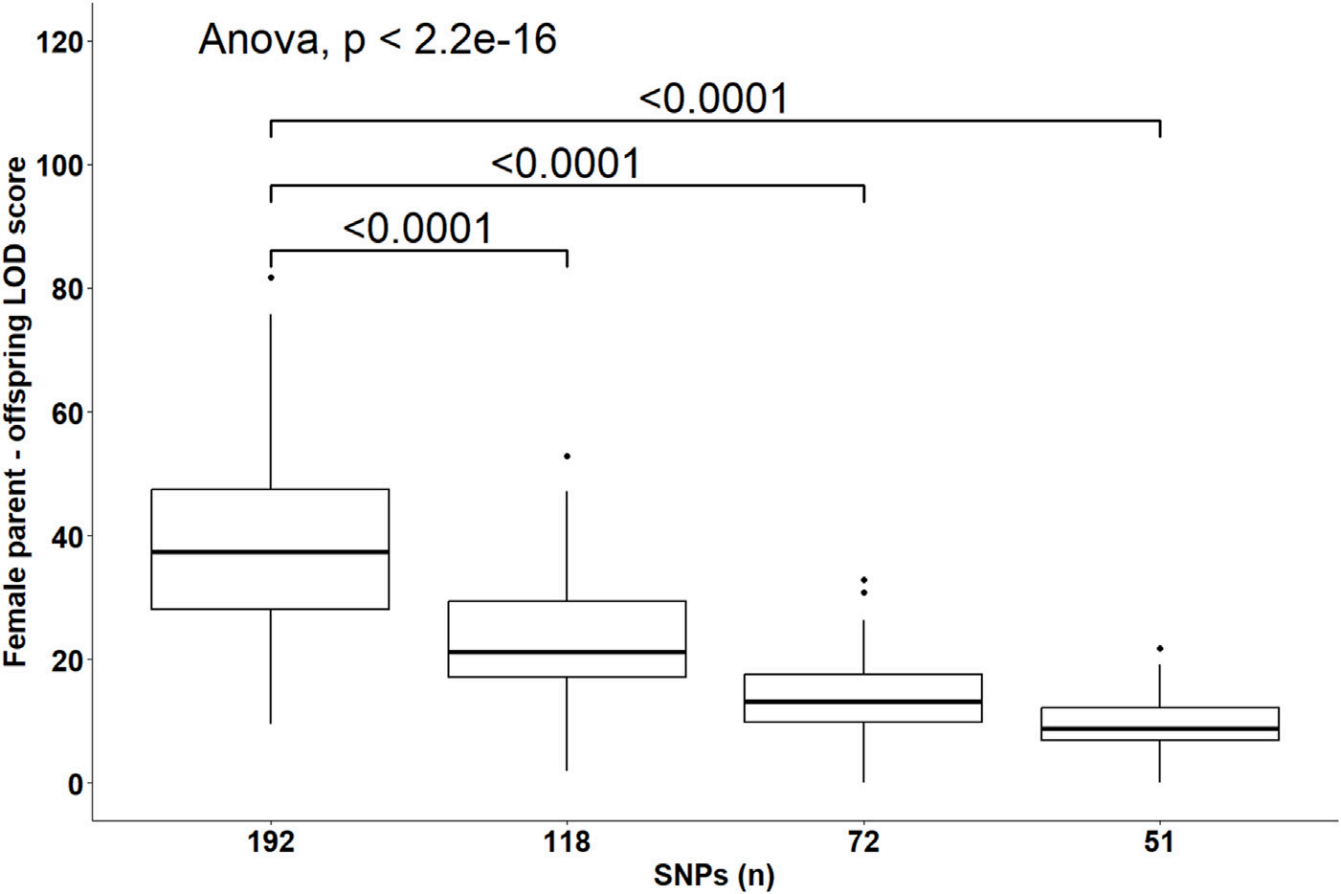
Distribution of the log-likelihood (LOD) scores of assignments to the female parent according to the number of SNPs included in the parentage assignment analysis. Brackets and their Bonferroni-corrected P-values indicate a significant difference in LOD scores in the 118, 72 and 51 SNPs datasets compared to the 192 SNPs dataset.

**FIGURE 10:**
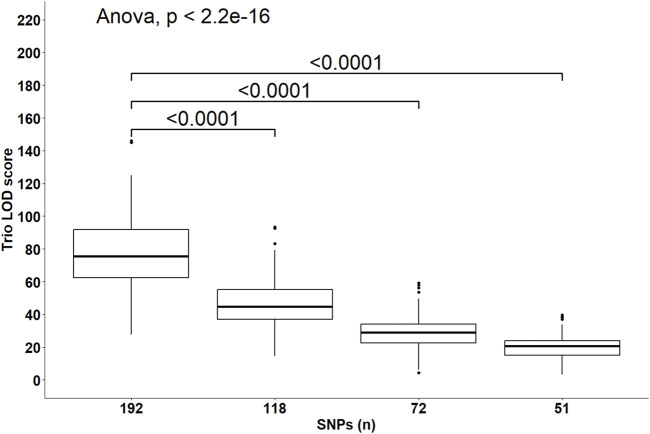
Distribution of the log-likelihood (LOD) scores of assignments to the parent pairs according to the number of SNPs included in the parentage assignment analysis. Brackets and their Bonferroni-corrected P-values indicate a significant difference in LOD scores in the 118, 72 and 51 SNPs datasets compared to the 192 SNPs dataset.

Despite the changes in C-LOD thresholds, *Cervus* accurately assigned 100% of tested larvae to their true male, female, and parent pairs, relative to the known pedigree. However, the A-LOD scores, for male-offspring (Figure 8), female-offspring (Figure 9), and parent pair-offspring (Figure 10) comparisons, decreased significantly as the number of SNPs was reduced from 192 to 51 (P < 0.0001).

### 3.4 Confidence level of parentage assignments

*Cervus* assigns a confidence level to each parentage assignment based on the C-LOD score threshold it meets. Table 4 presents the confidence levels for male parent-offspring assignments. With 192 SNPs, all assignments (100%) met the strict confidence threshold. At 118 SNPs, assignments meeting only the relaxed confidence threshold began to appear. When SNPs decreased to 72, unassigned most likely male parents started appearing, and at 51 SNPs, unassigned most likely male parents accounted for over 65% of assignments. A similar pattern was observed for female parent-offspring pairs (Table 5). For parent pair-offspring assignments (Table 6), they all (100%) met the highest confidence threshold in the two largest SNP subsets (192 and 118 SNPs). Assignments at the relaxed threshold began to occur with 72 SNPs, and unassigned most likely parent pairs appeared only with 51 SNPs. Simulated inbreeding levels had a marginal impact on assignment confidence, as shown in Table 4–6.

**TABLE 4:** Confidence levels of assignment to male parents according to the simulated inbreeding rate and the number of SNPs included in the parentage analyses. The data is the number of assignments in each confidence class expressed as a percentage of the total number of assignments (n = 75).

Inbreeding (%)	SNPs (n)	Confidence level^a^			
		Strict (95%)	Relaxed (80%)	Unassigned, most likely	Unassigned, not most likely
0	192	100.00	0.00	0.00	0.00
	118	78.67	21.33	0.00	0.00
	72	25.33	29.33	45.33	0.00
	51	13.33	4.00	81.33	1.33
5	192	100.00	0.00	0.00	0.00
	118	74.67	25.33	0.00	0.00
	72	38.67	18.67	42.67	0.00
	51	9.33	16.00	73.33	1.33
10	192	100.00	0.00	0.00	0.00
	118	78.67	21.33	0.00	0.00
	72	36.00	21.33	42.67	0.00
	51	8.00	21.33	69.33	1.33
25	192	100.00	0.00	0.00	0.00
	118	80.00	20.00	0.00	0.00
	72	36.00	25.33	38.67	0.00
	51	13.33	20.00	65.33	1.33
50	192	100.00	0.00	0.00	0.00
	118	85.33	14.67	0.00	0.00
	72	34.67	29.33	36.00	0.00
	51	12.00	21.33	65.33	1.33
100	192	100.00	0.00	0.00	0.00
	118	100.00	0.00	0.00	0.00
	72	36.00	52.00	12.00	0.00
	51	13.33	18.67	66.67	1.33

^a^ The confidence level can either be strict or relaxed. When the software *Cervus* is unable to assign a candidate parent or parent pair to an individual, it outputs the most likely parent or parent pair of the tested individual without assignment. When a most likely parent or parent pair is not available, *Cervus* outputs a parent or parent pair designated ‘not the most likely’.

**TABLE 5:** Confidence levels of assignment to female parents according to the simulated inbreeding rate and the number of SNPs included in the parentage analyses. The data is the number of assignments in each confidence class expressed as a percentage of the total number of assignments (n = 75).

Inbreeding (%)	SNPs (N)	Confidence level^a^			
		Strict (95%)	Relaxed (80%)	Unassigned, most likely	Unassigned, not most likely
0	192	100.00	0.00	0.00	0.00
	118	86.67	13.33	0.00	0.00
	72	48.00	14.67	36.00	1.33
	51	24.00	13.33	61.33	1.33
5	192	100.00	0.00	0.00	0.00
	118	81.33	18.67	0.00	0.00
	72	44.00	21.33	33.33	1.33
	51	17.33	18.67	62.67	1.33
10	192	100.00	0.00	0.00	0.00
	118	86.67	13.33	0.00	0.00
	72	41.33	26.67	30.67	1.33
	51	16.00	20.00	62.67	1.33
25	192	100.00	0.00	0.00	0.00
	118	86.67	13.33	0.00	0.00
	72	45.33	26.67	26.67	1.33
	51	13.33	25.33	60.00	1.33
50	192	100.00	0.00	0.00	0.00
	118	86.67	13.33	0.00	0.00
	72	42.67	33.33	22.67	1.33
	51	17.33	21.33	61.33	1.33
100	192	100.00	0.00	0.00	0.00
	118	100.00	0.00	0.00	0.00
	72	46.67	38.67	13.33	1.33
	51	14.67	24.00	60.00	1.33

^a^ The confidence level can either be strict or relaxed. When the software *Cervus* is unable to assign a candidate parent or parent pair to an individual, it outputs the most likely parent or parent pair of the tested individual without assignment. When a most likely parent or parent pair is not available, *Cervus* outputs a parent or parent pair designated ‘not the most likely’.

**TABLE 6:** Confidence levels of assignment to parent pairs according to the simulated inbreeding rate and the number of SNPs included in the parentage analyses. The data is the number of assignments in each confidence class expressed as a percentage of the total number of assignments (n = 75).

Inbreeding (%)	SNPs (N)	Confidence level^a^			
		Strict (95%)	Relaxed (80%)	Unassigned, most likely	Unassigned, not most likely
0	192	100.00	0.00	0.00	0.00
	118	100.00	0.00	0.00	0.00
	72	84.00	16.00	0.00	0.00
	51	30.67	34.67	30.67	0.00
5	192	100.00	0.00	0.00	0.00
	118	100.00	0.00	0.00	0.00
	72	84.00	16.00	0.00	0.00
	51	20.00	46.67	33.33	0.00
10	192	100.00	0.00	0.00	0.00
	118	100.00	0.00	0.00	0.00
	72	82.67	17.33	0.00	0.00
	51	8.00	58.67	30.67	0.00
25	192	100.00	0.00	0.00	0.00
	118	100.00	0.00	0.00	0.00
	72	81.33	18.67	0.00	0.00
	51	17.33	48.00	34.67	0.00
50	192	100.00	0.00	0.00	0.00
	118	100.00	0.00	0.00	0.00
	72	76.00	24.00	0.00	0.00
	51	18.67	40.00	41.33	0.00
100	192	100.00	0.00	0.00	0.00
	118	100.00	0.00	0.00	0.00
	72	70.67	29.33	0.00	0.00
	51	12.00	45.33	42.67	0.00

^a^ The confidence level can either be strict or relaxed. When the software *Cervus* is unable to assign a candidate parent or parent pair to an individual, it outputs the most likely parent or parent pair of the tested individual without assignment. When a most likely parent or parent pair is not available, *Cervus* outputs a parent or parent pair designated ‘not the most likely’.

## 4 Discussion

Implementing genetic selection in BSF could greatly benefit from genomic resources, such as SNP data, to develop tools like low-density marker panels. These panels could support genetic selection, enable PA-assisted pedigree-based selection, and improve genetic variability management in populations under selection. This study aimed to identify SNP subsets suitable for parentage assignment in both the laboratory and commercial BSF colonies sampled in this study. To achieve this, we used GBS, a reduced representation sequencing approach that offers a cost-effective alternative to whole genome sequencing. GBS protocols, especially when highly multiplexed, often result in moderate to low sequencing depth and increased missing data, which can impact genotype quality and marker availability for further analyses (Whalen et al., 2019). To address these limitations, we applied strict filtering criteria to retain only high-quality, informative SNP markers.

### 4.1 Population statistics and markers quality

The MAF distributions observed in the initial filtered SNP dataset provide support for a limited impact of ascertainment bias in this study. Across both colonies and three sampled generations, the MAF values exhibited a broad distribution without strong skewness toward rare or common alleles (Supplementary Figures S1–S3). This pattern indicates that the SNP discovery process captured a broad range of allele frequencies rather than being biased toward the most polymorphic loci, a common concern in SNP-based studies (Bradbury et al., 2011; Lachance and Tishkoff, 2013; Geibel et al., 2021). Nevertheless, given the stringent MAF filtering applied to produce the final SNP sets used for PA, long-term monitoring of marker informativeness will be necessary to ensure continued high-confidence assignment accuracy over successive generations.

The relatedness between the laboratory and commercial colonies was reflected in multiple population statistics despite 5 years of independent breeding. Differences in H_e_, H_o_, Δ_Het_, and Pi between colonies were small but statistically significant, likely due to consistent but subtle distributional shifts across many loci caused by differences in breeding practices, which have been shown to influence the genetic differentiation between BSF populations (Hoffmann et al., 2021). This degree of differentiation was reflected in the results of the PCA, which showed a degree of separation between colonies, and by the AMOVA, which revealed that 5.57% of the total genetic variance was attributable to differences between colonies (P = 0.001). These findings reflect the common origin of the laboratory and commercial subpopulation. The observed lower-than-expected heterozygosity and the observed inbreeding level likely result from our experimental design: in the first generation, parents were randomly selected from their source colonies, where prior inbreeding levels were unknown, while in subsequent generations, full-sib mating was used to maintain each of the 12 families per colony, thereby increasing inbreeding levels over time.

High-quality markers are essential for accurate parentage analysis. A previous study found that a MAF threshold of 0.5 provides the most power for PA, with minimal gains above a MAF of 0.40 (Anderson and Garza, 2006). Based on this finding, we divided our filtered SNPs into four subsets with a MAF ranging from >0.40 to >0.48, reducing SNP numbers from 192 to 51 due to the strict filtering criteria. Thus, the number of SNPs and the MAF were completely confounded in our study. Additionally, the strict filtering criteria led to the removal of all SNPs on the two smallest chromosomes of the BSF genome (LR899015 and NC035232).

The SNPs retained in all four subsets showed moderate genetic diversity, as indicated by the observed and expected heterozygosity. The PIC measures a marker’s ability to detect genetic differences, with higher values indicating greater informativeness for genetic analyses (Serrote et al., 2020), including PA. PIC values closer to 1.0 are highly informative, while values between 0.25 and 0.50 are somewhat informative (Serrote et al., 2020). In our study, the average PIC was around 0.40 across all SNP subsets, indicating slightly below average informativeness. This result was expected, as PIC values tend to decrease with increased inbreeding (Wang et al., 2020).

### 4.2 Parentage assignment rate and confidence

Although the heterozygosity and PIC of the four SNP subsets were moderate, they were sufficient to achieve perfect assignment accuracy, with 100% concordance between *Cervus*-assigned parents and the true known parents across all simulated inbreeding levels. However, achieving high-confidence assignments (i.e., assignments made at the strict 95% confidence level) required a greater number of SNPs. Specifically, as few as 118 SNPs were sufficient to assign larvae to their parent pairs with high confidence, whereas assigning larvae to an individual parent (either male or female) consistently at high confidence required the full set of 192 SNPs. A study on cattle and sheep using the opposing homozygotes method found that SNP requirements for PA varied by species, with at least 200 SNPs with a MAF between 0.35 and 0.45 needed to minimize false negatives, and 700 SNPs required to fully exclude false positives (Strucken et al., 2016). Similarly, a study on a commercial pig population using *Cervus*’ likelihood method showed that 60 SNPs enabled 100% correct assignment of 66 sires, though as the number of candidate sires per offspring included in the analysis increased to 304, at least 80 SNPs were needed for perfect assignment (Harlizius et al., 2011). In the same study, 80 or more SNPs were required to achieve 100% correct assignments to the sire as the number of sires increased because these sires included full and half sibs (Harlizius et al., 2011). This likely reflects the similarity of genotypes among related parents, which reduces exclusion probabilities and increases the need for more markers to ensure accurate PA in inbred populations (Harlizius et al., 2011).

Perfect assignment rates relative to known parents must be interpreted in the context of their confidence level. The lower the C-LOD score (between −999.0 and +999.0) and the higher the A-LOD scores, the more power a SNP set will have to correctly and confidently assign tested individuals to their candidate parents (Kalinowski et al., 2007; Marshall et al., 1998). In our study, simulations involving the largest SNP subset (n = 192 SNPs) yielded the lowest C-LOD scores (negative values) and assignments involving this same SNP set yielded the highest A-LOD scores, which led to a 100% rate of high confidence assignment of larvae to their male, female and parent pairs. As previously discussed, when candidate parents are related, more SNPs are required to correctly assign them (Harlizius et al., 2011). Consequently, as the number of SNPs in our SNP subsets decreased, the C-LOD scores increased towards positive values and A-LOD scores significantly decreased. Consequently, the assignment power of these SNP sets decreased leading to the occurrence of low confidence assignments. In the case of assignment to male or female parents individually, these low confidence assignments started occurring when the number of SNPs decreased to 118 SNPs. When smaller SNP subsets were used, unassigned parents were observed (>35% at 72 SNPs and >65% at 51 SNPs). However, it seems that considering the genotypes of both parents is associated with increased assignment power of SNP subsets as the combined maternal and paternal information imposes stricter genetic constraints on the offspring’s genotype, improving the discrimination between true and unrelated candidates and increasing the cumulative assignment likelihood. In the present study, even when using as few as 72 SNPs, more than 70% of the assignments were of high confidence when larvae were assigned to parent pairs. Unassigned, most likely parent pairs were only observed (>30%) when the number of SNPs included in the analyses decreased to 51 SNPs. It should be noted that the unassigned parents outputted by *Cervus* corresponded in all cases to the true parents of tested individuals which led to the 100% assignment rate discussed earlier. In practice, only high-confidence assignments should be considered because misassignment could dilute genetic progress or decrease genetic variability.

### 4.3 Challenges and future research

Before parentage assignment can be integrated into BSF breeding programs to implement pedigree-based selection, multiple challenges must be addressed.

Non-destructive DNA sampling remains a key challenge for the practical integration of genomic tools into *Hermetia illucens* breeding programs. In this study, destructive sampling methods were used to ensure DNA quantity and quality during the SNPs initial discovery phase. However, lethal sampling of selection candidates is incompatible with genetic selection. No research is yet available about non-lethal sampling in BSF, but several previous studies in insects provide promising models. In bumble bees, removing tarsal segments (the tips of mid- or hind-legs) yielded sufficient high-quality DNA for genotyping microsatellite markers without negatively impacting survival, foraging performance, mean body mass or reproductive success (Holehouse et al., 2003). Similarly, in dragonflies, mid-leg removal provided the highest DNA yield and quality, without compromised survival (Ožana et al., 2020). In small and fragile butterflies such as the Hermes copper, removal of a single leg allowed successful genotyping without adverse effects on lifespan or mating behavior (Marschalek et al., 2013). Wing clips could also serve as a non-lethal source of DNA. However, Ožana et al. (2020) found that wing-derived DNA was of low quality, and that clipping may negatively impact insect survival or reproductive fitness. Collectively, these studies highlight that leg removal is a minimally invasive and effective method across a range of insect taxa. It is thus reasonable to expect that removal of a leg, or even just the tarsus, would yield sufficient DNA for GBS or targeted SNP genotyping without affecting individual survival or reproductive potential. Future research should experimentally validate non-destructive sampling strategies in BSF by testing DNA yield and genotyping success from legs or other minimally invasive sources, and by monitoring post-sampling survival, reproductive output, and fitness traits. The approach based on leg or wing clips sampling assumes that selection decisions will be made at the adult stage. However, if selection is to be conducted earlier, at the larval stage, then such non-lethal sampling techniques are not applicable. In such cases, new protocols will need to be developed to enable non-destructive DNA extraction from live larvae, potentially through approaches such as environmental DNA (eDNA) sampling (*e.g.,* frass). Previous studies in aquatic organisms, including mollusks and early-stage fish larvae, have shown that eDNA released into surrounding water can be used for accurate and non-invasive genotyping (Holman et al., 2019; Espinoza et al., 2017). In fish larvae, this method enabled genotyping success rates of up to 98% for microsatellites and allowed for the unambiguous assignment of 98% of individuals to a specific dam and sire pair (Espinoza et al., 2017). In bivalve mollusks, Holman et al. (2019) found that 99% of correct SNP genotype calls were possible to achieve using eDNA. These findings raise the possibility that similar approaches could be adapted for BSF larvae, potentially by collecting DNA from larval substrate or frass. The suitability of exuviae as a non-invasive DNA source appears to be species-dependent. For example, Wang et al. (2022) demonstrated that exuviae from locusts yielded sufficient high-quality DNA for reliable PCR amplification and mutation screening, even enabling early, non-lethal identification of genome-edited individuals. In contrast, Ožana et al. (2020) reported that dragonfly exuviae provided DNA of insufficient quality and quantity for consistent genotyping, due in part to environmental contamination and minimal residual cellular material. These contrasting findings highlight the importance of species-specific validation before exuviae can be reliably adopted as a DNA source. In the context of BSF, future work should assess DNA yield and integrity across larval stages to determine whether exuviae are a viable option for genotyping in early selection schemes. In all cases, successful development of such methods would represent a critical step toward functional DNA-based pedigree reconstruction in BSF breeding programs.

Another critical challenge for the future application of SNP-based parentage assignment in *Hermetia illucens* is the potential limited transferability of SNP panels across genetically divergent populations or over multiple generations. SNP ascertainment bias, the preferential discovery of SNPs that are common in the initial sampling pool, can distort allele frequency distributions and artificially inflate measures of genetic diversity within the discovery population while reducing them in external populations (Bradbury et al., 2011; Lachance and Tishkoff, 2013; Geibel et al., 2021). In the present study, we attempted to mitigate the risk of ascertainment bias by randomly sampling the initial stock of larvae used to create the experimental population from the two colonies. Offspring larvae from subsequent generations were also randomly selected for DNA extraction. The MAF distribution in the initial pool of SNPs, from which the markers informative for parentage assignment were selected, showed no evidence of strong ascertainment bias across the two colonies or the three sampled generations (Supplementary Figures S1–S3). However, previous studies on BSF have shown that colonies can diverge rapidly due to strong founder effects, inbreeding, and artificial or natural selection (Rhode et al., 2020; Hull et al., 2024). These processes increase the risk that a SNP panel developed in one generational cohort may lose informativeness in subsequent generations or in other colonies, thus affecting the robustness of parentage assignment. Furthermore, rapid declines in heterozygosity and effective population size, a characteristic of mass-reared BSF colonies may exacerbate this loss of informativeness (Rhode et al., 2020; Hull et al., 2024). Consequently, SNP panel performance should be validated periodically across independent populations and over successive generations. Methods proposed for minimizing ascertainment bias in SNP array development (Lachance and Tishkoff, 2013; Geibel et al., 2021) could be adapted for BSF to design robust and transferable genotyping panels suited for breeding programs.

Another major challenge in the routine application of SNP-based parentage assignment in BSF is the turnaround time required for genotyping, from sample collection through DNA extraction, library preparation, sequencing, and data analysis. Although GBS pipelines offer high flexibility and marker discovery potential, they typically involve multi-step library preparation procedures and relatively long data processing times, which may limit the feasibility of selection in short-lived insects like BSF. Recent methodological advances offer promising solutions to reduce turnaround time and cost. The NanoGBS protocol (Torkamaneh et al., 2020) miniaturizes the traditional GBS library preparation by using acoustic droplet ejection (ADE) technology, reducing reaction volumes tenfold, handling time by 75%, library preparation costs by 67%, and genotyping costs by 72% per sample. This method enables the construction of high-quality libraries while requiring only 10 ng of input DNA, making it well-suited for BSF where non-lethal and minimal sampling are priorities. Furthermore, the genotyping-in-thousands by sequencing (GT-Seq) approach (Campbell et al., 2015) offers an alternative strategy for rapid and targeted genotyping of hundreds of SNPs. By using multiplex PCR and next-generation sequencing, GT-Seq can genotype thousands of individuals in a single sequencing lane at a very reduced cost per sample while dramatically simplifying library preparation. Both NanoGBS and GT-Seq methods represent attractive avenues for future implementation of cost-effective, high-throughput parentage assignment in BSF. Future research should explore the optimization of NanoGBS protocols specifically for BSF samples, including non-lethal tissue sources, and should evaluate the performance of GT-Seq panels developed from the informative SNP sets identified here. Combining these methods could make genotyping sufficiently rapid and affordable to support routine breeding decisions within a single generation cycle. While this study demonstrates the feasibility of using GBS-derived SNPs for parentage assignment in BSF, it is important to situate this step within the broader context of selection programs. Effective pedigree-based selection requires more than just timely genotyping. It also depends on obtaining reliable phenotypic data, estimating breeding values, and executing mating decisions within the biological constraints of the breeding cycle. In the case of BSF, traits of interest such as larval weight, protein content, or feed conversion require the development of automated phenotyping platforms to ensure scalability in commercial production.

## 5 Conclusion

In conclusion, our study demonstrates that genotyping-by-sequencing can generate genomic resources useful for parentage assignment in black soldier fly (*Hermetia illucens*) colonies. Our analyses showed that despite using an inbred experimental population, 118 to 192 informative markers were sufficient to achieve a 100% assignment rate at a 95% confidence level. The ability to track pedigrees in commercial settings through parentage assignment represents an important steppingstone for establishing breeding programs based on estimated breeding values that more accurately reflect the genetic potential of selection candidates. The list of markers identified in the present study can be used to develop a GT-seq panel that can be utilized to implement pedigree-based selection or to manage genetic diversity in BSF populations under selection. Future research should focus on developing non-lethal DNA sampling methods, optimizing rapid genotyping platforms such as NanoGBS or GT-seq, and validating panel robustness across generations and populations. Addressing these challenges will be critical to translating proof-of-concept successes into large-scale BSF breeding programs.

## Data Availability

The datasets presented in this study can be found in the NCBI Sequence Read Archive under BioProject number PRJNA1177819.
